# di-Cysteine Residues of the *Arabidopsis thaliana* HMA4 C-Terminus Are Only Partially Required for Cadmium Transport

**DOI:** 10.3389/fpls.2020.00560

**Published:** 2020-05-26

**Authors:** Stanislaus Antony Ceasar, Gilles Lekeux, Patrick Motte, Zhiguang Xiao, Moreno Galleni, Marc Hanikenne

**Affiliations:** ^1^InBioS – PhytoSystems, Functional Genomics and Plant Molecular Imaging, University of Liège, Liège, Belgium; ^2^InBioS – Center for Protein Engineering, Biological Macromolecules, University of Liège, Liège, Belgium; ^3^Melbourne Dementia Research Centre, Florey Institute of Neuroscience and Mental Health, The University of Melbourne, Parkville, VIC, Australia

**Keywords:** Arabidopsis, AtHMA4, Cadmium (Cd), Cd-binding, metal specificity, P-type ATPase

## Abstract

Cadmium (Cd) is highly toxic to the environment and humans. Plants are capable of absorbing Cd from the soil and of transporting part of this Cd to their shoot tissues. In Arabidopsis, the plasma membrane Heavy Metal ATPase 4 (HMA4) transporter mediates Cd xylem loading for export to shoots, in addition to zinc (Zn). A recent study showed that di-Cys motifs present in the HMA4 C-terminal extension (AtHMA4c) are essential for high-affinity Zn binding and transport *in planta*. In this study, we have characterized the role of the AtHMA4c di-Cys motifs in Cd transport *in planta* and in Cd-binding *in vitro*. In contrast to the case for Zn, the di-Cys motifs seem to be partly dispensable for Cd transport as evidenced by limited variation in Cd accumulation in shoot tissues of *hma2hma4* double mutant plants expressing native or di-Cys mutated variants of AtHMA4. Expression analysis of metal homeostasis marker genes, such as *AtIRT1*, excluded that maintained Cd accumulation in shoot tissues was the result of increased Cd uptake by roots. *In vitro* Cd-binding assays further revealed that mutating di-Cys motifs in AtHMA4c had a more limited impact on Cd-binding than it has on Zn-binding. The contributions of the AtHMA4 C-terminal domain to metal transport and binding therefore differ for Zn and Cd. Our data suggest that it is possible to identify HMA4 variants that discriminate Zn and Cd for transport.

## Introduction

Cadmium (Cd) is one of the most toxic substances present in the environment (Nriagu, 1988; Clemens et al., 2013) and represents a major health threat to humans, especially upon long-term exposure to low concentrations (Satarug et al., 2003, 2010). Although it may occasionally act as a prosthetic group in biologically active metalloproteins (Lane and Morel, 2000), Cd is usually considered to have no biological function.

Widespread low-level contamination of soil and water by Cd stems mostly from anthropogenic sources, e.g., mining, non-ferrous metal manufacturing, or the application of phosphate fertilizers and sewage sludge on soils (Clemens et al., 2013; Tóth et al., 2016). Although Cd emissions into the environment have declined in many industrialized countries, it is still prevalent in emerging countries and low-level contamination of soils results in important contamination of the food chain. This calls for a comprehensive understanding of the Cd accumulation mechanisms in plants (Clemens et al., 2013).

The plasma membrane Heavy Metal ATPase 4 (HMA4) belongs to P_IB_-type ATPases that transport heavy metal ions across cell membranes. Heavy Metal ATPase 4 is a main contributor to Cd xylem loading and root-to-shoot translocation in *Arabidopsis thaliana* (Arabidopsis) (Wong and Cobbett, 2009), although its primary function is to ensure, together with HMA2, sufficient zinc (Zn) supply to shoot tissues and to developing seeds (Mills et al., 2003; Hussain et al., 2004; Verret et al., 2004; Wong and Cobbett, 2009; Cun et al., 2014). An *hma2hma4* double mutant has a stunted growth phenotype, resulting from severe shoot Zn deficiency (Hussain et al., 2004). HMA4 is also a key factor in the Zn and Cd hyperaccumulation syndrome found in a number of calamine species such as *Arabidopsis halleri* or *Noccaea caerulescens*, i.e., species that can establish populations on zinc/cadmium/lead-rich, so-called calamine soils (Hanikenne et al., 2008; Krämer, 2010; Merlot et al., 2018).

Typical of P_IB_-type ATPases, the HMA4 protein possesses eight transmembrane domains that play a key role in ion selectivity (Argüello, 2003; Argüello et al., 2007; Smith et al., 2014; Wang et al., 2014; Lekeux et al., 2019), multiple cytoplasmic catalytic domains, and N- and C-terminal cytosolic extensions rich in putative metal-binding amino acids such as Cys, His, Asp, and Glu (Williams and Mills, 2005; Argüello et al., 2007; Rosenzweig and Argüello, 2012). Hence, in the Arabidopsis HMA4 (AtHMA4) protein, a Cys–Cys–Thr–Ser–Glu motif in the N-terminal domain binds one Zn(II) atom with high affinity, and this interaction is essential for the function of the protein *in planta* (Zimmermann et al., 2009; Laurent et al., 2016). The long cytosolic C-terminal extension of AtHMA4 (AtHMA4c, 470 amino acids), which usually lacks in its bacterial homologs, exhibits 13 di-Cys motifs and an 11 His-stretch and is capable of binding 10-11 Zn(II) atoms with high affinity (Bækgaard et al., 2010; Lekeux et al., 2018). While the function of the His-stretch remains elusive (Verret et al., 2005; Lekeux et al., 2018), the di-Cys motifs were recently shown to confer nanomolar affinity for Zn(II) and to play a key role in the protein’s function in Zn homeostasis *in planta* (Lekeux et al., 2018).

Although AtHMA4 is known to transport Cd in addition to Zn (Talke et al., 2006; Courbot et al., 2007; Wong and Cobbett, 2009), the role of AtHMA4c in Cd binding and transport has not been determined. It was, however, shown that expression of AtHMA4c alone in yeast conferred Cd tolerance (Bernard et al., 2004). Here, we examined the functional role of the multiple di-Cys motifs in AtHMA4c in Cd transport *in vivo* and Cd(II) binding properties *in vitro*. We show that, in contrast to the case of Zn, the di-Cys motifs are only partially required for Cd transport to shoot tissues and only partially contribute to the Cd(II)-binding capacity of the C-terminal domain of AtHMA4. Our data suggest that the contribution of the AtHMA4 C-terminal domain to metal transport differs for Zn and Cd.

## Materials and Methods

### Plant Material and Growth Conditions

*Arabidopsis thaliana* L. Heynhold (accession Columbia, Col-0) and *hma2hma4* double mutant *A. thaliana* plants (Col-0 background) (Hussain et al., 2004) were used in all experiments. *hma2hma4* mutant plants expressing either a native *AtHMA4* or an *AtHMA4CCAA* (13 di-Cys→13 di-Ala motifs) variant under the control of the *AtHMA4* promoter were reported previously (Lekeux et al., 2018). Sterile seeds were germinated for 14 days under short days (22°C and 8 h day^–1^ photoperiod) on 1/2 Murashige and Skoog (MS) agar medium containing 1% sucrose. These two-week-old seedlings were then transferred in hydroponics [Araponics growth trays, Araponics, Belgium (Tocquin et al., 2003)] and supplied with modified Hoagland medium containing 1 μM ZnSO_4_ (control condition) and grown for another 3 weeks in short days (Talke et al., 2006; Charlier et al., 2015; Nouet et al., 2015). The Cd treatment was initiated from the 4^th^ week by supplying 0.05 or 1 μM CdSO_4_, still in the presence of 1 μM ZnSO_4_. Nutrient solutions were changed weekly. After 3 weeks of Cd treatment, root and shoot tissues were harvested separately for elemental profiling and gene expression analysis. All experiments were conducted in three biological replicates, each including two independent T3 homozygous lines per genotype, representative of the lines described in Lekeux et al. (2018).

### Elemental Profiling

Metal content in root and shoot tissues was analyzed as described (Lekeux et al., 2018).

### Gene Expression Analysis

Total RNAs were isolated from shoot and root samples using the NucleoSpin RNA Plant Kit (Macherey-Nagel GmbH & Co., Germany). The samples were treated on column with DNAse. cDNAs were then synthesized using 1 μg of total RNA, oligo-dT and the RevertAid H Minus First Strand cDNA Synthesis Kit (Thermo Fisher Scientific, United States). Quantitative RT-PCR reactions were performed in 384-well plates with a QuantStudio 5 Real-Time PCR system (Thermo Fisher Scientific, United States) using Takyon Low Rox SYBR MasterMix (Eurogentec, Belgium). Three technical replicates were performed as described (Lekeux et al., 2018) for each sample/primer combinations. The quality of the quantitative PCR was checked by inspection of dissociation and amplification curves. For each primer pair (Supplementary Table S1), mean reaction efficiencies were calculated using the LinRegPCR software (Ruijter et al., 2009) and then used to quantify relative gene expression levels by normalization using three reference genes (At1g18050, *UBQ10*, and *EF1*α) (Czechowski et al., 2005) with the qBase software (Biogazelle; Hellemans et al., 2007). The adequacy of the reference genes to normalize gene expression in the experimental conditions was checked using the geNorm module in qBase (Vandesompele et al., 2002).

### Cadmium Binding Assay

The protocol and experimental conditions for the analysis of Cd(II) binding to the AtHMA4 C-terminal domain (AtHMA4c as wild-type and AtHMA4CCAAc as mutant versions) were as described in Lekeux et al. (2018) except for the use of the Mag-Fura-2 (MF2) probe (Invitrogen M1290) instead of the Par probe. The binding affinity of Par for Cd(II) is too weak to allow a quantitative analysis (Kocyła et al., 2015). The metal-free MF2 probe exhibits an absorbance maximum at 366 nm with ε = 2.99 × 10^4^ M^–1^ cm^–1^ which was used in this work to quantify MF2 concentration (Zimmermann et al., 2009). It was reported that MF2 can bind one equivalent of Cd(II) with a dissociation constant *K*_D_ = 126 nM and Cd(II) binding to MF2 led to a decrease in absorbance at 366 nm until the probe was saturated with one equivalent of Cd(II) (de Seny et al., 2001). Consequently, in this work, Cd(II) binding assay was performed by titration of the MF2 probe (16.2 μM) with CdCl_2_ (taken from a 200 μM stock) in the presence of a target HMA4 protein domain (3.3–3.4 μM) with the titration process monitored by the absorbance change at 366 nm. Likewise, the concentration of the CdCl_2_ stock used in this work was calibrated by the control titration of the MF2 probe by setting the titration turning point to Cd(II)/MF2 = 1.0. The experiments were conducted in MOPS buffer (50 mM, pH 7.3, 100 mM NaCl) with inclusion of TCEP (1 mM) to maintain the reduced protein form. Cd(II) binding stoichiometry and affinity to each protein domain were estimated by a quantitative comparison of the titration curve to the control titration curve without protein under the same condition (see section “Results and Discussion” for details). The protein samples were produced and purified in fusion to the Maltose-Binding Protein (MBP) as described (Lekeux et al., 2018).

### Data Analysis

Data were analyzed, and statistics were performed using SPSS software.

## Results and Discussion

### The di-Cys Motifs of the AtHMA4 C-Terminal Domain Are Only Partially Required for Cd Transport

**FIGURE 1:**
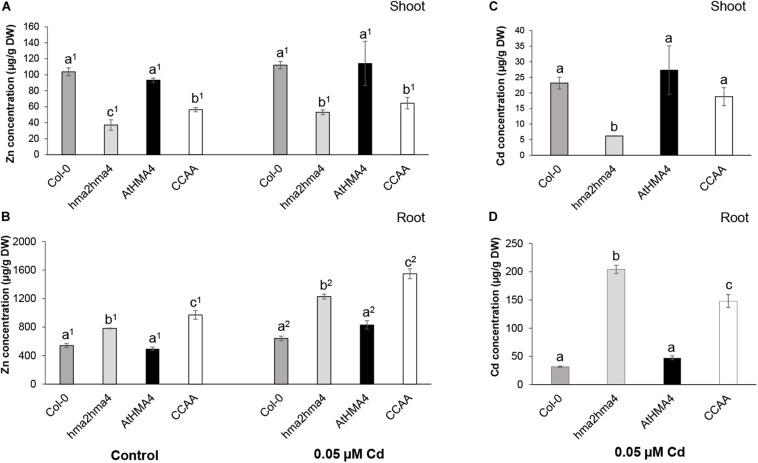
Zinc and cadmium accumulation in shoot **(A,C)** and root **(B,D)** tissues of wild-type (Col-0), *hma2hma4* mutant, and *hma2hma4*-expressing AtHMA4 or AtHMA4CCAA plants upon hydroponic growth in control conditions (1 μM Zn, no Cd) or exposed for 3 weeks to 0.05 μM Cd. Values (in μg g^–1^ DW) are means ± SD of three biological replicates each consisting of pools of three plants from two independent lines per genotype. Statistically significant differences (*P* < 0.05) between genotypes within treatments (one-way ANOVA followed by Tukey’s multiple comparison test) or between treatments within genotypes (*t*-test for independent means) are indicated by different letters or different superscript numbers, respectively. DW, dry weight; CCAA, di-Cys → di-Ala motifs.

To examine the function of the C-terminal domain of AtHMA4 (AtHMA4c) in Cd transport, *hma2hma4* double mutant plants expressing the native *AtHMA4* or a *AtHMA4CCAA* (13 di-Cys→13 di-Ala motifs) variant under the control of the *AtHMA4* promoter (Lekeux et al., 2018) were assessed for Cd accumulation. Col-0 and *hma2hma4* double mutant plants were also assessed as wild-type and mutant controls, respectively. In the AtHMA4CCAA variant, all 13 di-Cys motifs found in the AtHMA4c domain were mutated into di-Ala motifs (Lekeux et al., 2018). Both native and mutant protein variants were shown to localize to the plasma membrane and to be expressed at similar levels (Lekeux et al., 2018). When grown in control conditions (1 μM Zn), the *hma2hma4* mutant displayed a typical phenotype with lower shoot and higher root Zn accumulation compared to wild-type plants (Figures 1A,B; Hussain et al., 2004; Nouet et al., 2015; Lekeux et al., 2018). Whereas expression of the native AtHMA4 fully restored this phenotype, expression of AtHMA4CCAA resulted in only limited complementation indicating impaired Zn transport to shoot tissues, as described (Lekeux et al., 2018).

As Cd treatment, a low Cd concentration (0.05 μM Cd) was selected so as to enable Cd accumulation in tissues without causing toxicity, which may have triggered spurious effects on metal homeostasis and general plant fitness (Nouet et al., 2015; Lekeux et al., 2019). The Zn accumulation pattern in tissues of the different genotypes was only marginally altered upon Cd treatment, with a noticeable, but barely significant, increase in Zn root accumulation in all genotypes (Figures 1A,B).

Upon Cd treatment (0.05 μM Cd), the *hma2hma4* mutant displayed strongly increased Cd accumulation in roots (∼6.5-fold) and reduced Cd accumulation in shoots (∼3.7-fold), compared to wild-type plants. This phenotype was fully reversed by the expression of the native AtHMA4 in the mutant background (Figures 1C,D). However, in contrast to the case of Zn, the *hma2hma4* double mutant plants expressing the AtHMA4CCAA variant displayed shoot Cd concentrations similar to wild-type plants (Figure 1C). Cadmium accumulation in root tissues of the AtHMA4CCAA plants was intermediate between the high and low Cd concentrations accumulated in the *hma2hma4* and wild-type plants, respectively (Figure 1D), suggesting that Cd transport capacity of the AtHMA4CCAA is somehow impacted by the di-Cys motif mutation. While the shoot/root Zn concentration ratio was only marginally increased (1.2-fold) upon expression of AtHMA4CCAA in the *hma2hma4* background, the shoot/root Cd concentration ratio was increased 4.2-fold, indicative of a higher Cd transport to shoots. Similar results were obtained when plants were exposed to a higher, toxic, Cd concentration (1 μM Cd, Supplementary Figure S1). All genotypes displayed reduced growth and chlorosis (data not shown). Zn shoot accumulation remained low in AtHMA4CCAA-expressing plants, whereas Cd shoot accumulation was significantly higher in AtHMA4CCAA plants compared to the *hma2hma4* mutant (Supplementary Figure S1). These observations are striking as they suggest that while the di-Cys motifs present in the AtHMA4 C-terminal domain are key to enable Zn transport (Lekeux et al., 2018), they are at least partly dispensable for Cd transport to the shoot.

Finally, the plant genotype or the Cd treatment had limited effect on the accumulation of other elements in tissues (Supplementary Table S2). For instance, the iron (Fe) concentration was about 20% lower in shoots of the *hma2hma4* double mutant and AtHMA4CCAA plants compared to wild-type plants upon Cd exposure.

### The Expression of Metal Homeostasis Marker Genes Is Altered in Response to Cd

**FIGURE 2:**
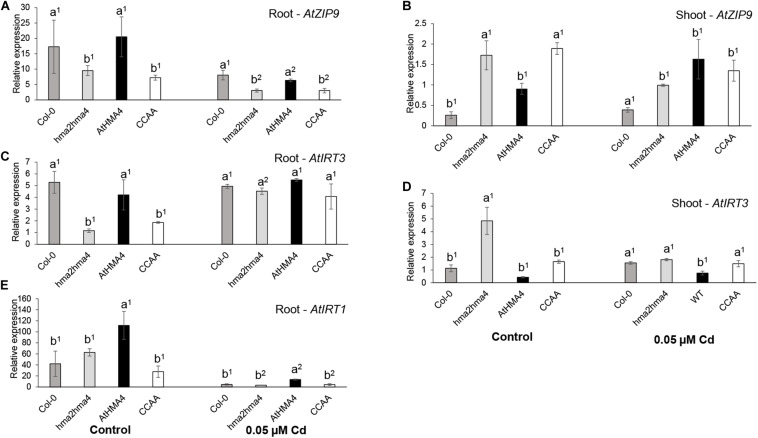
Expression analysis of metal homeostasis genes. *AtZIP9* **(A,B)**, *AtIRT3* **(C,D)**, and *AtIRT1* **(E)** relative expression levels were measured in root **(A,C,E)** and shoot **(B,D)** tissues of wild-type (Col-0), *hma2hma4* mutant, and *hma2hma4*-expressing AtHMA4 or AtHMA4CCAA plants upon hydroponic growth in control conditions (1 μM Zn, no Cd) or exposed for 3 weeks to 0.05 μM Cd. Relative expression values are means ± SEM of three biological replicates each consisting of pools of three plants from two independent lines per genotype. Statistically significant differences (*P* < 0.05) between genotypes within treatments (one-way ANOVA followed by Tukey’s multiple comparison test) or between treatments within genotypes (*t*-test for independent means) are indicated by different letters or different superscript numbers, respectively. CCAA: di-Cys → di-Ala motifs.

To further assess the physiology of the plants, the expression levels of metal-responsive genes were assessed and compared in root (*AtIRT3*, *AtZIP9*, and *AtIRT1*) and shoot (*AtIRT3* and *AtZIP9*) tissues of plants grown in both control and Cd-treated (0.05 μM) conditions (Figure 2).

First, *AtZIP9* and *AtIRT3* are Zn-responsive genes, induced upon Zn deficiency and repressed upon Zn excess (Talke et al., 2006). The two genes are regulated by the bZIP19 and bZIP23 transcription factors, which are key regulators of the Zn deficiency response in Arabidopsis (Assunção et al., 2010; Castro et al., 2017). As shown previously (Nouet et al., 2015; Lekeux et al., 2018), *AtZIP9* was strongly responding to Zn deficiency in the shoot of the *hma2hma4* mutant, whereas both genes were repressed in roots compared to the wild-type (Figures 2A–D). Again, the expression of AtHMA4CCAA in the *hma2hma4* background did not complement this phenotype (Lekeux et al., 2018). Cd exposure had contrasting effects on the expression of *AtZIP9* and *AtIRT3*. Indeed, Cd reduced the expression of *AtZIP9* in root tissues of all genotypes although the pattern of differential expression between wild-type and mutant plants remained visible (Figure 2A). Conversely, Cd induced the expression of *AtIRT3* in root tissues of *hma2hma4* and AtHMA4CCAA plants and among genotype differences were no longer observed (Figure 2B). This induction may be responsible for higher Zn in roots under Cd supply (Figure 1B and Supplementary Table S2). In shoot tissues, the Cd treatment overall reduced differences of *AtIRT3* and *AtZIP9* expression among genotypes (Figures 2B,D). The observed differences in expression of *AtIRT3* and *AtZIP9* in response to Cd are unlikely to be responsible for the contrasting Cd accumulation pattern between the *hma2hma4* mutant and the other genotypes (Figures 1C,D), but rather reflect their different contribution to metal homeostasis adjustment in response to Cd. Indeed, Cd may compete for uptake with other divalent cations, such as Zn or Fe. *AtIRT3*, encoding a Zn and Fe transporter (Lin et al., 2009), has been shown to be regulated by Zn deficiency (Talke et al., 2006; van de Mortel et al., 2006), but not by Fe deficiency (Buckhout et al., 2009; Yang et al., 2010). In contrast, *AtZIP9* is regulated by both Zn and Fe deficiency (Talke et al., 2006; Yang et al., 2010), under the control of bZIP19 mostly (Inaba et al., 2015), and FIT, a transcription factor with a major role in regulating the Fe deficiency response (Colangelo and Guerinot, 2004), respectively.

Second, *AtIRT1* encodes the main Fe uptake transporter at the root epidermis in Arabidopsis (Vert et al., 2002; Thomine and Vert, 2013). AtIRT1 has low ion selectivity and is also responsible for the uptake of additional divalent metal cations such as Zn or Cd (Vert et al., 2002; Barberon et al., 2011). AtIRT1 is subjected to a complex regulation by Fe availability but also by the presence of its other metal substrates, at both transcriptional and post-translational levels enabling fine-tuning of metal uptake (Connolly et al., 2002; Barberon et al., 2011; Barberon and Geldner, 2014; Dubeaux et al., 2018). In particular, *AtIRT1* expression is down-regulated upon Cd exposure at both transcript and protein levels (Connolly et al., 2002). Recent work suggested that differential transcription levels as well as functionality of IRT1 may contribute to among-population variation in Zn and/or Cd accumulation in the metal hyperaccumulators *A. halleri* (Corso et al., 2018; Schvartzman et al., 2018) and *N. caerulescens* (Halimaa et al., 2019). Altogether, there is ample evidence that IRT1 represents a major route of entry for Cd in plant tissues. Therefore, *AtIRT1* expression was assessed here in root tissues of all genotypes in both control and Cd conditions to determine whether the differential Cd accumulation observed among genotypes (Figures 1C,D) resulted from changes in *AtIRT1* expression. Upon Cd treatment, *AtIRT1* expression was down-regulated in roots of all genotypes compared to control conditions (Figure 2E) and there was very little variation among wild-type and mutant genotypes, except for the slightly higher expression of *AtIRT1* in *hma2hma4* lines complemented by the native AtHMA4 which is difficult to interpret. These observations suggested that higher Cd accumulation in shoots of AtHMA4CCAA plants compared to the *hma2hma4* mutant did not result from IRT1-driven differential Cd root uptake.

### Mutating the di-Cysteine Residues in the AtHMA4 C-Terminus Only Partially Alters Cd Binding

**FIGURE 3:**
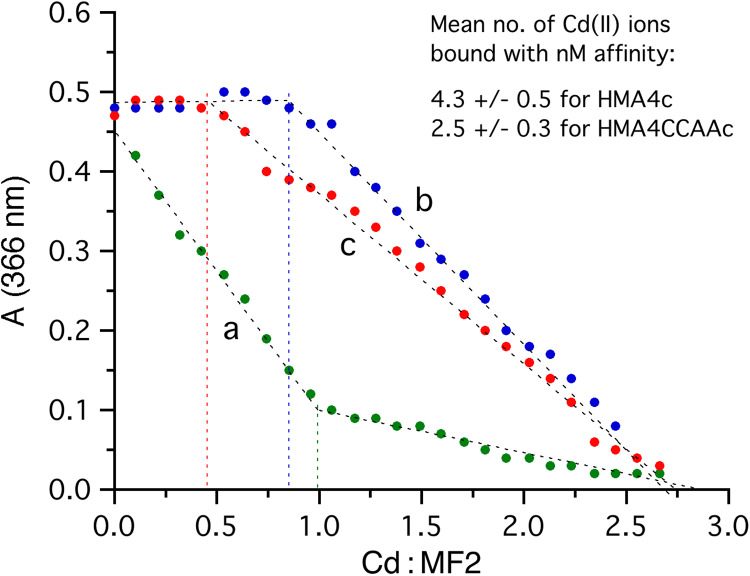
Cd(II)-binding assay for the AtHMA4 C-terminal domains by competition with MF2 probe in MOPS buffer (pH 7.3, 100 mM NaCl, 1 mM TCEP): curve (a) titration of MF2 (16.2 μM) with CdCl_2_ (200 μM); curve (b) titration of a mixture of MF2 (16.2 μM) and MBP-AtHMA4c (3.4 μM) with CdCl_2_ (200 μM); curve (c) titration of a mixture of MF2 (16.2 μM) and MBP-AtHMA4CCAAc (3.3 μM) with CdCl_2_ (200 μM). The apparent turning point for each titration curve is indicated by a vertical dashed line. The number of Cd(II) ions bound to each protein domain with affinities out-competing that of MF2 was estimated by the product of the molar ratio Cd(II)/MF2 at the titration turning point and the molar ratio MF2/protein in the titration mixture. Values in the chart represent data from one experiment representative of three independent experiments, whereas values in the table insert are means ± SD of the three independent experiments.

To link the above *in vivo* observations to the possible impact of the di-Cys motifs in AtHMA4c to the Cd(II) binding property, Cd(II) binding assays were performed by titrations of the purified MBP-AtHMA4c or MBP-AtHMA4CCAAc fusion proteins with Cd^2+^ in the presence of the Cd(II)-responding probe MF2 (de Seny et al., 2001; Zimmermann et al., 2009). A control titration of Cd^2+^ into a MF2 solution (16.2 μM) without protein component led to a steady decrease in absorbance at 366 nm with an apparent turning point at Cd(II)/MF2 = 1.0 (Figure 3, curve a), consistent with the formation of a 1:1 Cd(II)-MF2 complex with the reported sub-micromolar affinity (*K*_D_ = 126 nM) (de Seny et al., 2001). Inclusion of the purified MBP carrier protein in the same MF2 solution had little effect on the titration curve (data not shown), demonstrating that MBP lacked detectable Cd(II) binding sites by the MF2 probe. However, an equivalent titration with substitution of MBP by MBP-AtHMA4c (3.4 μM) in the same MF2 probe solution generated a very different titration curve (compare curve b to curve a in Figure 3). With titration of Cd^2+^, the solution absorbance at 366 nm remained essentially unchanged until a molar ratio Cd(II)/MF2 = 0.9 ± 0.1 was reached for three independent titrations (Figure 3, curve b), indicating that the AtHMA4c protein domain out-competed MF2 for high-affinity Cd(II) binding before the titration turning point and can bind 4.3 ± 0.5 equivalents of Cd(II) with high affinities estimated at the nanomolar concentration range (ie, *K*_D_ < < 126 nM). The titration curve (b) after the turning point was somewhat flatter than the titration curve (a) before the titration turning point (Figure 3), suggesting the existence of further weaker Cd(II) binding sites with affinities comparable to that of MF2 at *K*_D_ = 126 nM.

On the other hand, another equivalent titration with inclusion of MBP-AtHMA4CCAAc (3.3 μM) instead of MBP-AtHMA4c led to a titration curve with an apparent turning point at a lower Cd(II)/MF2 of 0.50 ± 0.06 (Figure 3, curve c), suggesting that the AtHMA4CCAAc protein domain can bind 2.5 ± 0.3 equivalents on average of Cd(II) with nanomolar affinity. However, the titration curve (c) after the turning point was further flattened, suggesting that AtHMA4CCAAc featured somewhat much weaker Cd(II)-binding sites than did AtHMA4c. Overall, mutation of the 13 di-Cys motifs in the AtHMA4 C-terminal domain has led to a loss of ∼42% of high-affinity Cd(II) binding. This is in contrast to previous Zn-binding assays which showed that the CCAA mutations reduced high-affinity Zn(II) binding at *K*_D_ < 1 nM from ∼7 equivalents for AtHMA4c to only ∼2 equivalents for AtHMA4CCAAc, i.e., a loss of ∼70% high-affinity Zn(II) binding (Lekeux et al., 2018).

## Conclusion

Cd(II) binding by AtHMA4c was thus lower than Zn(II) binding, with ∼4.3 equivalents for Cd(II) on average (Figure 3) and ∼11 equivalents for Zn(II) [7 with *K*_D_ < 1 nM and 4 with *K*_D_ = 1–10 nM (Lekeux et al., 2018)]. In the absence of the di-Cys motifs, the AtHMA4CCAAc domain retains 2.5 equivalents (>50%) of high-affinity Cd(II) binding which might result from either other non-di-Cys ligands such as single Cys and/or numerous His, Asp, and Glu residues also present in AtHMA4CCAAc. Alternatively, the retained Cd(II) binding in AtHMA4CCAAc might result from creation of new high-affinity binding sites caused by structural coordination rearrangement induced upon removal of di-Cys motifs, which may be facilitated by the fact that the AtHMA4c domain is intrinsically disordered. This remaining Cd(II)-binding capacity of AtHMA4CCAAc appeared to be sufficient, at low Cd(II) exposure, to at least partially enable Cd(II) transport and translocation to the plant shoot (Figure 1), whereas it was shown recently that the di-Cys motif of AtHMA4c is essential for Zn transport (Lekeux et al., 2018). In the past, several attempts have been made to identify mutations enabling metal transporters to discriminate between essential metals (e.g., Fe or Zn) and non-essential toxic metals (e.g., Cd) (Rogers et al., 2000; Pottier et al., 2015; Liedschulte et al., 2017), with the aim of favoring transport of specific metals for biofortification. For HMA4-related proteins in tobacco, modification of the protein sequence to reduce Cd accumulation in tobacco leaves resulted in drastically altered Zn homeostasis and consequently strongly impaired growth (Liedschulte et al., 2017). In contrast, plants expressing an AtHMA4 variant with a substitution of Phe_177_, present in transmembrane domain 2, into a Leu displayed significantly decreased Cd accumulation in shoots while Zn accumulation was maintained to a level enabling normal growth of Arabidopsis (Lekeux et al., 2019). Here, the contribution of the multiple di-Cys motifs in the AtHMA4 C-terminal domain to metal transport appears to differ for Zn and Cd, which may be instrumental to discriminate between the two metals for biofortification or phytoremediation strategies.

## Data Availability Statement

All datasets generated for this study are included in the article/Supplementary Material.

## Author Contributions

MH and MG conceived and directed the study. MH, MG, SC, GL, and ZX designed the experiments. SC and GL performed the experiments. SC, MH, MG, and ZX analyzed the data. MH, MG, and PM contributed reagents, materials, and analysis tools. SC, MH, ZX, and MG wrote the manuscript. All authors commented on the manuscript.

## Conflict of Interest

The authors declare that the research was conducted in the absence of any commercial or financial relationships that could be construed as a potential conflict of interest.
