# Supplementary material for: di-Cysteine Residues of the Arabidopsis thaliana HMA4 C-Terminus Are Only Partially Required for Cadmium Transport
Source: Front Plant Sci. 2020 May 26;11:560. doi: 10.3389/fpls.2020.00560 (PMC7264368; doi:10.3389/fpls.2020.00560)
Supplement: Supplementary file 1 [file Data_Sheet_1.docx]

**Table S1.** Sequences and reaction efficiencies of quantitative RT-PCR primer pairs.

| **Gene** | **Primer** | **Sequence 5'=>3'** | **Reaction efficiency** | **Standard deviation RE** |
| --- | --- | --- | --- | --- |
| *At1g18050* | Fwd | CCATTCTACTTTTTGGCGGCT | 1.964 | 0.020 |
|  | Rev | TCAATGGTAACTGATCCACTCTGATG |  |  |
| *UBQ10* | Fwd | GGCCTTGTATAATCCCTGATGAATAAG | 1.936 | 0.028 |
|  | Rev | AAAGAGATAACAGGAACGGAAACATAGT |  |  |
| *AtEF1α* | Fwd | TGAGCACGCTCTTCTTGCTTTCA | 1.939 | 0.023 |
|  | Rev | GGTGGTGGCATCCATCTTGTTACA |  |  |
| *AtZIP9* | Fwd | CCATCACTACTCCGATCGGTGT | 1.869 | 0.018 |
|  | Rev | CACCAATGCTGCAACGCTATAA |  |  |
| *AtIRT3* | Fwd | AGTCATCCTCCTGGTCATGATT | 1.967 | 0.023 |
|  | Rev | GAGCATGACCAATGTCGAT |  |  |
| *AtIRT1* | Fwd | CCCCGCAAATGATGTTACCTT | 1.951 | 0.024 |
|  | Rev | GGTATCGCAAGAGCTGTGCAT |  |  |

RE: reaction efficiency

**Table S2.** Elemental profiling of the root and shoot tissues of wild-type (Col-0), *hma2hma4* mutant and *hma2hma4* expressing AtHMA4 or AtHMA4CCAA plants upon growth in control conditions (Ctrl, 1 µM Zn, no Cd) or exposed for 3 weeks to 0.05 µM Cd. Values (in µg/g DW) are mean±SD of 3 biological replicates each consisting of pools of 3 plants from 2 independent lines per genotype. The data were analyzed with one-way ANOVA followed by Tukey’s multiple comparison test. Statistically significant differences (P<0.05) between means within tissues and treatments are indicated by different letters. DW: dry weight; CCAA: di-Cys --> di-Ala motifs; n.d.: not detected.

| **Samples** | **Ca** | **Cd** | **Cu** | **Fe** | **K** | **Mg** | **Mn** | **Mo** | **Zn** |
| --- | --- | --- | --- | --- | --- | --- | --- | --- | --- |
| **Ctrl - Shoot** |  |  |  |  |  |  |  |  |  |
| Col-0 | 41590 ±1856^a^ | n.d. | 36±1.5^a^ | 99±9.0^a^ | 48807±3231^a^ | 7011±178^a^ | 181±16.3^a^ | 9±6.2^a^ | 104±4.8^a^ |
| *hma2hma4* | 45193±1170^b^ | n.d. | 41±1.0^a^ | 98±5.5^a^ | 45016±5449^b^ | 7220±157^b^ | 200±12.3^a^ | 30±6.1^b^ | 37±6.5^c^ |
| AtHMA4 | 45932±2080^b^ | n.d. | 27±0.7^b^ | 77±4.6^b^ | 49394±1303^a^ | 7042±287^a^ | 205±16.5^a^ | 7±4.5^a^ | 93±2.6^a^ |
| AtHMA4CCAA | 47750±3246^c^ | n.d. | 25±1.7^b^ | 88±7.3^a^ | 47674±2205^a^ | 7041±395^a^ | 219±22.0^a^ | 3±3.0^a^ | 56±2.5^b^ |
|  |  |  |  |  |  |  |  |  |  |
| **Ctrl - Root** |  |  |  |  |  |  |  |  |  |
| Col-0 | 7964±602^a^ | n.d. | 192±8.9^a^ | 2041±145^a^ | 32105±3721^a^ | 2453±82^a^ | 108±12.4^a^ | 71±15^a^ | 540±28^a^ |
| *hma2hma4* | 7060±122^a^ | n.d. | 251±3.1^b^ | 1932±24.2^a^ | 53266±6708^b^ | 2590±77^b^ | 75±6.7^a^ | 175±2.6^b^ | 782±5.7^b^ |
| AtHMA4 | 6529±234^a^ | n.d. | 229±15^b^ | 1677±67^a^ | 39177±2593^a^ | 2418±37^a^ | 167±19.9^b^ | 85±9.6^a^ | 495±23^a^ |
| AtHMA4CCAA | 6986±764^a^ | n.d. | 222±25^b^ | 1833±186^a^ | 48340±2555^b^ | 2729±139^c^ | 292±48.8^c^ | 89±11.4^a^ | 971±61^c^ |
|  |  |  |  |  |  |  |  |  |  |
| **0.05 µM Cd - Shoot** |  |  |  |  |  |  |  |  |  |
| Col-0 | 46605±1639^a^ | 23±1.9^a^ | 35±2.1^a^ | 99±9.4^a^ | 48865±2057^a^ | 7317±204^a^ | 178±13.1^a^ | 21±4.3^a^ | 112±4.6^a^ |
| *hma2hma4* | 54468±2999^b^ | 6±0.1^b^ | 45±0.2^b^ | 79±0.6^b^ | 48844±971^a^ | 7848±315^b^ | 206±14.1^a^ | 11±5.9^b^ | 53±3.0^b^ |
| AtHMA4 | 55345±15176^b^ | 27±7.8^a^ | 43±10^b^ | 99±8.7^a^ | 51688±12559^b^ | 8599±2360^c^ | 215±63^a^ | 31±8.7^c^ | 114±27^a^ |
| AtHMA4CCAA | 53038±3049^b^ | 19±2.9^c^ | 34±4.2^a^ | 80±5.6^b^ | 45200±2177^c^ | 7651±456^b^ | 226±15.7^a^ | 27±2.9^c^ | 65±7.2^b^ |
|  |  |  |  |  |  |  |  |  |  |
| **0.05 µM Cd - Root** |  |  |  |  |  |  |  |  |  |
| Col-0 | 6428±155^a^ | 32±1.1^a^ | 211±7.3^a^ | 1827±111^a^ | 48655±2553^a^ | 2213±65^a^ | 110±4.1^a^ | 129±14^a^ | 640±32^a^ |
| *hma2hma4* | 9037±333^b^ | 204±7.0^b^ | 256±34^a^ | 3129±270^b^ | 27152±9779^b^ | 2605±263^a^ | 50±8.3^b^ | 139±11^a^ | 1229±34^b^ |
| AtHMA4 | 8369±397^b^ | 47±3.4^a^ | 243±6.5^a^ | 2707±309^b^ | 47657±4467^a^ | 2589±187^a^ | 110±6.7^a^ | 109±23^b^ | 830±58^a^ |
| AtHMA4CCAA | 7451±383^b^ | 148±11^b^ | 204±13.5^a^ | 2681±196^b^ | 39013±2558^a^ | 2700±80^a^ | 172±40.2^c^ | 126±6.2^a^ | 1548±69^c^ |


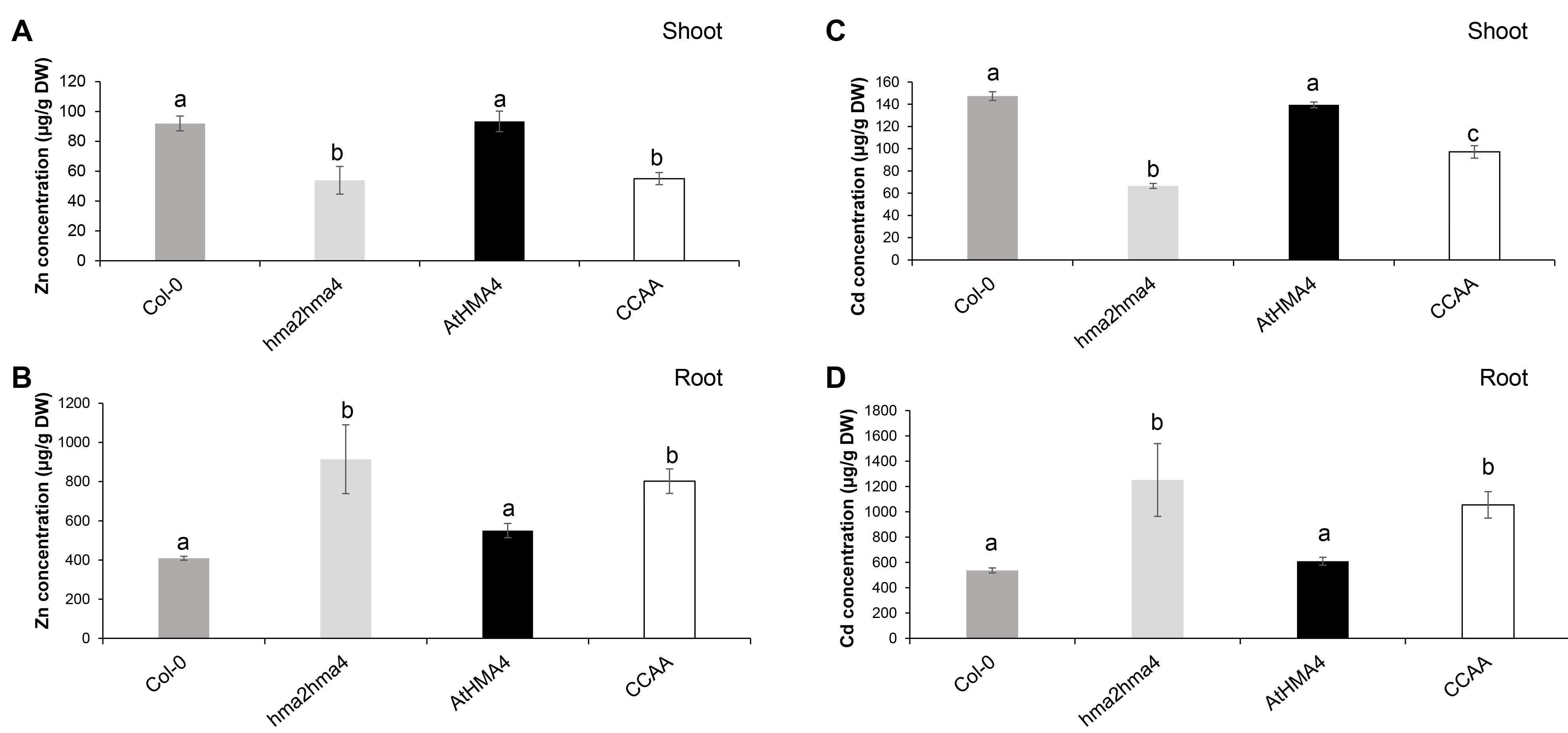


**Figure S1.** Zinc and cadmium accumulation in shoot (A, C) and root (B, D) tissues of wild-type (Col-0), *hma2hma4* mutant and *hma2hma4* expressing AtHMA4 or AtHMA4CCAA plants upon hydroponic growth exposed for 3 weeks to 1 µM Cd. Values (in µg/g DW) are means ± SD of 3 biological replicates each consisting of pools of 3 plants from 2 independent lines per genotype. The data were analyzed with one-way ANOVA followed by Tukey’s multiple comparison test. Statistically significant differences (P<0.05) between means are indicated by different letters. DW: dry weight; CCAA: di-Cys --> di-Ala motifs.
